# Early inpatient rehabilitation for acutely hospitalized older patients: a systematic review of outcome measures

**DOI:** 10.1186/s12877-019-1201-4

**Published:** 2019-07-09

**Authors:** Patrick Heldmann, Christian Werner, Nacera Belala, Jürgen M. Bauer, Klaus Hauer

**Affiliations:** 1 Network Aging Research (NAR), Heidelberg University, Bergheimer Str. 20, 69115 Heidelberg, Germany; 2 Agaplesion Bethanien Hospital Heidelberg, Geriatric Center at the Heidelberg University, Heidelberg, Germany; 3 Center for Geriatric Medicine, Heidelberg University, Heidelberg, Germany

**Keywords:** Acute care, Hospitalization, Aged, Rehabilitation, Exercise, Outcome measures

## Abstract

**Background:**

Selecting appropriate outcome measures for vulnerable, multimorbid, older patients with acute and chronic impairments poses specific challenges, which may have caused inconsistent findings of previous intervention trials on early inpatient rehabilitation in acutely hospitalized older patients. The aim of this review was to describe primary outcome measures that have been used in randomized controlled trials (RCTs) on early rehabilitation in acutely hospitalized older patients, to analyze their matching, and to evaluate the effects of matching on the main findings of these RCTs.

**Methods:**

A systematic literature search was conducted in PubMed, Cochrane CENTRAL, CINAHL, and PEDro databases. Additional studies were identified through reference and citation tracking. Inclusion criteria were: RCT, patients aged ≥65 years, admission to an acute hospital medical ward (but not to an intensive medical care unit), physical exercise intervention (also as part of multidisciplinary programs), and primary outcome measure during hospitalization. Two independent reviewers extracted the data, assessed the methodological quality, and analyzed the matching of primary outcome measures to the intervention, study sample, and setting. Main study findings were related to the results of the matching procedure.

**Results:**

Twenty-eight articles reporting on 24 studies were included. A total of 33 different primary outcome measures were identified, which were grouped into six categories: functional status, mobility status, hospital outcomes, adverse clinical events, psychological status, and cognitive functioning. Outcome measures differed considerably within each category and showed a large heterogeneity in their matching to the intervention, study sample, and setting. Outcome measures that specifically matched the intervention contents were more likely to document intervention-induced benefits. Mobility instruments seemed to be the most sensitive outcome measures to reveal such benefits.

**Conclusions:**

This review highlights that the selection of outcome measures has to be highly specific to the intervention contents as this is a key factor to reveal benefits attributable to early rehabilitation in acutely hospitalized older patients. Inappropriate selection of outcome measures may represent a major cause of inconsistent findings reported on the effectiveness of early rehabilitation in this setting.

**Trial registration:**

PROSPERO CRD42017063978.

**Electronic supplementary material:**

The online version of this article (10.1186/s12877-019-1201-4) contains supplementary material, which is available to authorized users.

## Background

Older patients treated in hospital - and those who treat them - face complex challenges which arise from a multitude of negative health conditions. In addition to acute medical illness as the cause of the hospital admission and the high prevalence of multimorbidity in this patient population, older patients frequently show further associated geriatric conditions, such as malnutrition, cognitive impairment, delirium, impairments in (instrumental) activities of daily living ([I]ADL), incontinence, and sensory impairment [1]. Apart from the fact that each of these conditions will request a specific, often enough individualized response, the mass of negative conditions, and the advanced frailty status frequently observed in these patients, put them at an extraordinary risk for hospital-associated deconditioning. As an expected consequence, the prevalence of functional decline during hospital stay is high, varying from 30 to80% depending on the assessment methodology, medical status, and age cohorts included [2, 3]. The consequences of this decline during are manifold, ranging from re-hospitalization, nursing home placement [4], and subsequent mortality [5] to an increased number of falls, poor quality of life, and increased use of health-related resources [6].

For all patients admitted to acute medical care, the subsequent phase of immobilization is crucial as it will drastically impair their functional status to a level where autonomy is seriously endangered [7]. Consequently, hospital admission represents a vulnerable period in the treatment process in which an early onset of rehabilitation and physical training is of utmost importance, providing the basis for post-recovery and subsequent therapeutic and rehabilitative care.

The effect of early physical exercise interventions in acutely hospitalized older patients has already been examined in a number of previous systematic reviews [3, 8–13], reporting heterogeneous results across different outcomes and outcome categories such as hospital outcomes, adverse clinical events, or functional and mobility outcomes. A potential cause of this inconclusive evidence for the benefits of early physical exercise interventions has been addressed in one of these reviews, hypothesizing that the adaption level of the intervention to the capabilities of the patients might have played a critical role for the effectiveness of such interventions in acutely hospitalized older patients [13]. However, contrary to this hypothesis, patient-tailored physical exercise interventions were not found to be superior to those interventions that were not. Another potential cause for the still limited evidence might be the use of various outcome measures, which has been reported in most of the aforementioned reviews [3, 10, 11, 13]. However, none of these reviews specifically addressed the heterogeneity and the appropriateness of the outcome measures selected in the previous studies. The selection of the outcome measure(s), i.e. the operationalization of the outcome, is a critical step in designing a valid and useful clinical study [14]. In absence of an appropriate outcome measure, the impact of an intervention may be lost and benefits of the intervention may not be captured [14, 15]. Outcome measures used in clinical trials seem to have been most frequently evaluated focusing only on their psychometric properties [16, 17]. However, such focus fails to address also important questions about the suitability of the measures for their intended use. When reviewing and selecting an appropriate outcome measure for a tailored study design, the evaluation of the psychometric properties represents a first step, but also further requirements have to be considered. Most importantly, researchers should select outcome measures that match the intervention contents and specifically address the areas being targeted by them. If an intervention content is not well represented in the outcome measure, true changes in the relevant areas the researches expect to be influenced by the specific intervention may be lost because the selected outcome measure was unable to capture it. Further, it is important to determine whether the outcome measures are feasible in the target population. Feasibility aspects such as floor effects, indicating an overtaxation of patients, and ceiling effects, indicating an insufficient test challenge, must be considered, especially in the acute hospital setting with a highly heterogeneous patient population. Another criterion that must be considered when selecting appropriate outcome measures is to determine whether any features of the items could be problematic for use in the research setting. For example, IADL scales include items that assess an individual’s ability to perform instrumental home or community activities such as housekeeping and going shopping, which cannot be appropriately assessed within the acute care hospital setting [14, 18]. Meeting these requirements in the early hospital-based geriatric rehabilitation poses a particular challenge based on the fact, that acutely hospitalized older patients represent a heterogeneous, multimorbid and vulnerable patient population in a complex environment during a critical phase of recovery [9]. Consequently, potential multiple goals in the treatment of these patients will go along with different intervention strategies and outcome measures to be amalgamated into a specifically tailored study design, which may not have been achieved in previous studies.

The aim of this systematic review was (1) to describe outcome measures as used in previous intervention trials for early rehabilitation in acutely hospitalized older patients and analyze their matching to the contents of the intervention, the study sample, and the acute care hospital setting, and (2) to evaluate the effects of matching on the main findings reported in these intervention trials.

## Methods

### Search strategy and study selection

A systematic literature search was conducted in the electronic databases of PubMed, Cochrane CENTRAL, CINAHL, and PEDro from inception to December 2016. An extensive search strategy was developed for the PubMed database (Additional file 1: Table S1) and adjusted to the other electronic databases. Manual searching was also performed to identify additional studies by scanning reference lists of relevant review articles and included articles.

The inclusion criteria were as follows: (1) randomized, controlled intervention trial (RCT), (2) in older people aged 65 years or older (or 95% of participants aged at least 65 years), (3) admitted to an acute hospital medical ward but (4) not to an intensive medical care unit, (5) with a physical exercise intervention or a multidisciplinary program with physical exercise as a training component, both performed in an acute hospital medical ward, and (6) at least one primary outcome measure during acute care hospitalization. Studies were excluded if they were conducted in subacute hospital settings (e.g. rehabilitation wards), feasibility studies, or written in languages other than English.

The selection process was conducted following the methodology as described in the method guidelines of the Cochrane Collaboration [19]. Each step of the selection process was performed independently by two researchers (PH, NB), and disagreements were resolved by consensus or third party consultation (KH, JMB). The review followed the PRISMA guidelines for reporting systematic reviews and meta-analyses (see Additional file 2 for the completed PRISMA checklist [20]) and was registered at the PROSPERO International prospective register of systematic reviews (PROSPERO 2017: CRD42017063978).

### Data extraction

Data extraction was completed by the two reviewers (PH, NB) using a standardized data collection form as recommended by the Cochrane Collaboration [21]. For each study, the following data were extracted: author, country, sample characteristics, primary and secondary outcome measures during hospitalization, time point of measurement, intervention contents, and main findings on primary outcome measures. The extracted data were structured into a table and systematically analyzed.

### Data analysis

#### Matching of outcome measures

An initial set of guidelines to help evaluate the matching of outcome measures for clinical trials have been proposed by Coster (2013) [14]. Taking these guidelines into account, the primary outcome measures identified for each study during hospitalization were matched with the intervention contents, the sample included in the study, and the acute care hospital setting. The criteria used for this matching procedure were provided in Table 1. The matching procedure was performed independently by two researchers (PH, CW), and any disagreements were resolved by consensus or third party consultation (KH, JMB).

**Table 1:** Criteria for the matching of an outcome measure with the intervention, study sample, and setting

Criteria		Rating	
Intervention	Did the outcome measure match an intervention content?	“Match”	The outcome measure specifically addressed the exercise intervention or an intervention content of the multidisciplinary program (e.g., 6-Meter Walking Test → treadmill walking training; discharge destination → discharge planning).
		“Limited match”	The outcome measure addressed the exercise intervention or an intervention content of the multidisciplinary program only to a limited extent and/or included only single items that specially matched to the intervention (e.g., Barthel Index [transfer, mobility, and stairs items] → strengthening and mobility exercises; physical activity monitoring → weight-bearing exercises)
		“No match”	The outcome measure did not directly address the exercise intervention or an intervention content of the multidisciplinary program or the construct of the outcome measure was not addressed in the intervention (e.g., Lawton IADL scale → no IADL training content or discharge destination → additional exercise intervention).
Study sample	Was the outcome measure feasible in the study sample?	“Match”	The outcome measure showed no floor or ceiling effects (continuous outcomes) or represented no rare event (dichotomous outcomes). Ceiling and floor effects were defined as (1) ≥ 15% of participants reaching a score within the best or worst 15% of the instrument’s rating scale [22] or (2) when the mean score of the sample was within the best or worst 15% of the rating scale. Rare events were defined when the incidence of a dichotomous outcome (e.g., falls, mortality) was ≤15% in the sample.
		“No match”	The outcome measure showed floor or ceiling effects (continuous outcomes) or represented a rare event (dichotomous outcomes).
	How high was the missing data rate for the outcome measure in the study sample?	“Match”	The outcome measure had an acceptable missing data rate. Missing data included any outcome data that (1) could not be collected for reasons other than death or study withdrawal or (2) were collected but not presented. A missing data rate of < 15% was considered as acceptable.
		“No match”	The outcome measure did not have an acceptable missing data rate (≥ 15%).
Setting	Did the outcome measure match the acute care hospital setting?	“Match”	The outcome measure addressed a construct or activities that can be appropriately assessed within the acute care hospital setting (e.g., hospital costs or Barthel Index).
		“Limited match”	The outcome measure addressed a construct or activities that can be appropriately assessed only to a limited extent within the acute care hospital setting and/or included only single items or contents that were appropriate for use within the acute care hospital setting (e.g., combined ADL-IADL measures).
		“No match”	The outcome measure addressed a construct or activities that cannot be appropriately assessed within the acute care hospital setting (e.g., IADL measures).

The main findings reported on the primary outcomes were subsequently related to the results of the matching procedure, with special focus on the matches between the outcome measures and the intervention contents, representing the most important factor to demonstrate the impact on the relevant areas being targeted by an intervention [14]. The evaluation of the intervention effects was based on the significance level of between-group differences in the primary outcomes. *P*-values ≤0.05 were considered statistically significant.

#### Quality rating

Each included study was assessed using the PEDro scale, which consists of 11 items for rating the methodological quality of RCTs [23]. When available, confirmed PEDro scores from the PEDro database were used for the quality rating [24]. If no confirmed PEDro score was available, the quality rating was performed independently by two researchers (PH, NB). Disagreements were resolved by consensus or third party consultation (KH, JMB). A study with a PEDro score of ≤5 points is considered to be of low methodological quality at high risk of bias [25].

## Results

**Fig. 1:**
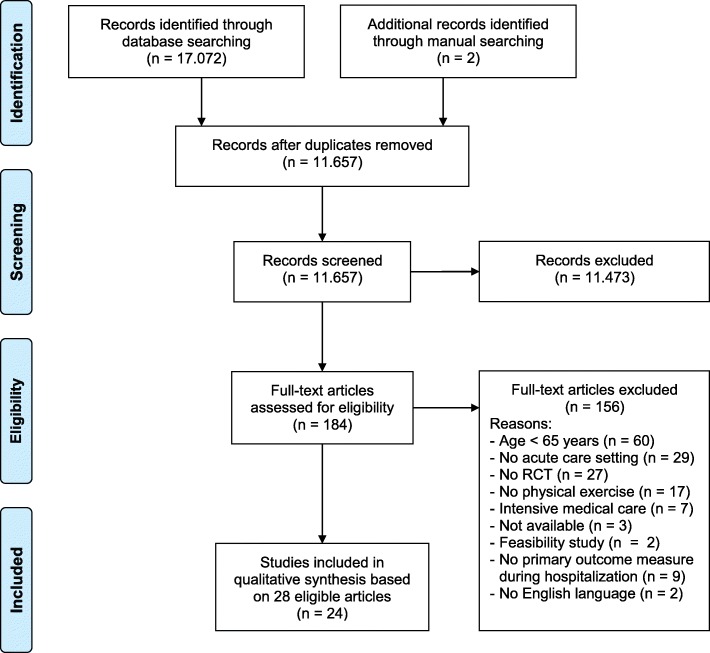
PRISMA flow chart of study selection

The search strategy yielded 17.074 potentially relevant articles (Fig. 1). After removing duplicates and screening of title and/or abstract, 184 articles were obtained in full text and evaluated for eligibility based on the predefined inclusion criteria. In total, 28 articles published between 1995 and 2016 were identified for inclusion. As four [26–29] and another two included articles [30, 31] reported each on the same RCT, the search finally resulted in 24 identified studies. The detailed data extracted for each of these studies were presented in Table 2.

**Table 2:** Characteristics of the included studies

Study Country	Sample	Intervention	Outcome measures during hospitalization* (category: outcome measure) *primary outcome measure in bold	Time point of measurement (primary outcome)	Main findings
Abizanda 2011 [32] Spain	*n* = 400 Mean age, 84 yrs. Females: *n* = 227 (57%); Patients with acute medical illness (stroke, cardiopulmonary pathologies, or other diagnoses)	Intervention: - Additional occupational therapy by special trained therapists (daily 45-min sessions, 5 days/week) - Day 1: physical, functional, cognitive social and emotional assessment; preparation of individual therapeutic plan - Day 2 until discharge: cognitive exercises, ADL training (mobility in bed, sitting and standing, chair to bed transfers, wheelchair to bed/toilet transfers, dressing, bathing, personal hygiene, toilet use) - Day of discharge: a second 30-min session in addition to the regular 45-min daily intervention; instruction for relatives or caregivers; recommendations for ADL at home Control: Conventional treatment with usual physiotherapy	**FCT: Barthel Index** (improvement of ≥10 pt. from admission to discharge) COG: CAM	Admission Discharge	Between-group differences at discharge: - Improvement in Barthel Index of ≥10 pt. from admission to discharge: n.s. (total sample, stroke/cardiopulmonary patients), ↑ (others) - Absolute improvement in Barthel Index: n.s. (total sample, stroke patients, others), ↑ (cardiopulmonary patients) Feasibility: - Missing data: Barthel Index = 0% (admission), 6% (discharge)
Blanc-Bisson 2008 [33] France	*n* = 76 Mean age: 85 yrs. Females: *n* = 55 (72%); Patients with acute medical illness	Intervention: - Additional early physiotherapy (start: day 1 or 2, 2 times/day for 30 min, 5 days/week), - Focus on leg extension exercises in the upright position - Nutritional supplements Control: - Walking with/without technical assistance or human help (start: day 3 to 6, 3 times/week until discharge) - Nutritional supplements - Physical therapy at home for 1 month	MOB: Handgrip strength (handheld dynamometry) **FCT: Katz ADL Index** BPN: Body weight, energy intake, protein intake, calf and arm circumferences, triceps skin fold, biochemical measures (serum albumin, C-reactive protein)	Admission Clinical stable condition	Changes from admission to clinical stable situation in total sample (time effect): - Katz ADL Index: ↓ Feasibility: - Missing data: Katz ADL Index = 0%
Brown 2016 [34] USA	*n* = 100 Mean age: 74 yrs. Females: *n* = 3 (3%) Patients with acute medical illness	Intervention: - Additional mobility protocol: Starting with basic transfers with progress to ambulation if tolerated (2 times/day, 15–20 min, 7 days/week) - Patients were encouraged to walk at each session - Physical activity behavioral strategy: goal setting, diary and interview to increase times out of bed Control: Usual care (physical therapy had to be ordered by physicians)	**FCT: Modified Katz ADL Index** HU: LOS, physical therapy ordered during hospitalization ACE: Falls	Admission Discharge	Between-group differences at discharge: - Modified Katz ADL Index: n.s. Changes during hospitalization in total sample: - Katz ADL Index: n.s. Group × time interaction during hospitalization: - Katz ADL Index: n.s. Feasibility: - Katz ADL Index: mean admission score in both groups was within the best 15% of the rating scale → ceiling effect
Czyzewski 2013 [35] Poland	*n* = 34 Mean age: 76 yrs. Females: *n* = 14 (41%); Patients with major abdominal surgery	Intervention: - Usual care with a modified exercise component based on the Proprioceptive Neuromuscular Facilitation concept (30 min/day) Control: Usual care (30 min/day)	**MOB: 10MWT, TUG** **FCT: Lawton IADL scale** **MOB: UCLA scale, PPSA** BPN: Forced ventilation capacity, first-second forced expiratory volume, maximal expiratory flow (spirometry) HU: LOS	3 days prior surgery 4 days after surgery	Within-group changes from 3 days prior surgery to 4 days after surgery: - 10MWT, TUG: ↓ in both groups - Lawton IADL scale, UCLA, PPSA: NA Between-group differences 4 days after surgery: - PPSA: ↑ - 10MWT, TUG: NA Feasibility: - Lawton IADL scale: mean admission score of the sample was within the best 15% of the rating scale → ceiling effect - Missing data (3 days prior & 4 days after surgery): 10MWT, TUG = 9%, SAP = 0%, UCLA, IADL: NA
Eyres 2005 [36] Australia	*n* = 15 Mean age: 80 yrs. Females: *n* = 9 (60%); Patients with acute medical illness	Intervention: - Daily additional occupational therapy - Self-care program (ADL), IADL training (e.g., cooking, laundry, café visits), community mobility (e.g., walking outdoors) Control: Usual care	**FCT: FIM** **PSY: Self-Efficacy Gauge, Life Satisfaction Index** HU: LOS, use of allied health services, use of community services, discharge destination	Admission Discharge	Within-group comparisons over time: - FIM ↑ (IG, CG) - Self-Efficacy Gauge: n.s. (IG, CG) - Life Satisfaction Index: n.s. (IG, CG) Feasibility: - Missing data: FIM, Self-Efficacy Gauge, Life Satisfaction Index = 0%
Hagsten 2004 [37] Sweden	*n* = 100 Mean age: 80 yrs. Females: *n* = 80 (80%); Patients with hip fracture	Intervention: - Additional occupational therapy (40–60 min, 5 days/week) - Self-care, independence at home (transfers, bathroom visits, morning activities, dressing), use of aids - Home visits - Instruction of a physiotherapist CG: Usual care from nursing staff, instruction of a physiotherapist	**FCT: Modified Klein-Bell ADL Scale (75 items of 4 areas: dressing, toilet visits, mobility, bathing/hygiene); mDRI with visual analogize scales for ADL, indoor IADL, and outdoor IADL** PSY: Study-specific mDRI items on fear of performing (I)ADL and for pain level during (I)ADL performance	Discharge	Between-group differences at discharge: - Modified Klein-Bell ADL scale: dressing ↑, toilet visits ↑, hygiene ↑, mobility: n.s. mDRI: ADL, indoor/outdoor IADLs, fear, pain: n.s. Feasibility: - Missing data: Klein-Bell ADL scale, mDRI = 0%
He 2015 [38] China	*n* = 101 Mean age: 71 yrs. Females: *n* = 11 (12%) Patient with acute COPD exacerbation	Intervention: - Patient education (physical activity behavior intervention): benefits and importance of daily exercise, pacing and energy-conservation technique to manage ADL - Stretching, endurance & strength training (endurance lower limb: walking with treadmill; upper limb: shoulder flexion and abduction with light weight; strength training: free weights or body weights) - breathing exercise: relaxation, breathing control, pursed-lip breathing, pacing during exercise - 30 min 2 times/day Control: Usual care	**MOB: 6MWT** **DS: mMRC dyspnea grade, ADL-Dyspnea scale, CRQ-SAS, CAT** Borg dyspnea scale, Bode index BPN: Resting/exercise oxygen saturation (spirometry, arterial blood gas analysis)	Admission Discharge	Within-group differences from admission to discharge: - 6MWT: ↑ (IG), n.s. (CG) - mMRC dyspnea grade: ↑ (IG), n.s. (CG) - ADL-Dyspnea scale: ↑ (IG), n.s. (CG) - CRQ-SAS: ↑ (IG), n.s. (CG) - CAT: ↑ in both groups Feasibility: NA
Jeffs 2013 [39] Australia	*n* = 649 Mean age: 79 yrs. Females: *n* = 340 (52%) Patients with acute medical illness	Intervention: - Graded physical activity and orientation program twice daily in addition to usual care - Physical activity program: progressive, variable resistance training against gravity, body or light weight (progression whenever a patient could perform 10 repetitions), - Cognitive exercise program: Orientation, (7 questions for improving orientation [day, month, year, date, ward, bed number, name of primary nurse]); - 2 times/day, 5 days/week, 20–30 min until discharge + self-training on weekends Control: Usual care (including: 24 h nursing care, daily medical assessment, allied health referral)	**COG: Number of delirious patients**, severity/duration of delirium (CAM) HU: Discharge destination, LOS	Admission Every 48 h until discharge	Between-group differences - Number of delirious patients: n.s. Feasibility: - No delirium in 94% of patients → rare event
Jones 2006 [40] Australia	*n* = 160 Mean age: 82 yrs. Females: *n* = 92 (58%) Patients with acute medical illness	Intervention: - Additional exercise program (2 times/day, 30 min) - Strengthening and mobility exercises (e.g., sit-to-stand transfer) specifically designed to be carried out in a hospital setting Control: Usual care with standard physiotherapy	**FCT: Barthel Index** MOB: TUG HU: Discharge destination, LOS ACE: Falls, mortality, deterioration in medical status	Admission Discharge	Between-group differences at discharge: - Barthel Index: n.s. Multivariable regression analyses: - Barthel Index: low admission Barthel Index & IG assignment = independent predictors of improving Barthel Index Feasibility: - Missing data: Barthel Index = 0%
Kimmel 2016 [41] Australia	*n* = 92 Mean age: 81 yrs. Females *n* = 59 (64%) Patients with hip fracture	Intervention: - Two additional physiotherapy sessions aimed to improve the functional advances achieved during the usual physiotherapy session (3 times/day, 7 days/week) Control: Usual care (physiotherapy: 1 time/day, 7 days/week)	**MOB: mILOAS**, TUG HU: LOS, Discharge destination, opioid equivalence score ACE: Postoperative complications PSY: Self-developed pain scale	Day 5	Between-group differences at post-operative Day 5: - mILOAS: n.s. Between-group differences controlled for confounding factors: - mILOAS: ↑ Feasibility: - Missing data: mILOA = 0%
Nikolaus 1999 [42] Germany	*n* = 545 Mean age, 81 yrs. Females: *n* = 400 (73%); Patients with acute medical illness	Intervention 1:Comprehensive geriatric assessment and interdisciplinary intervention in the hospital and at home, physical and occupational therapy (washing, eating, dressing, walking) twice a week up to twice a day for 30 min Intervention 2: Comprehensive geriatric assessment with recommendation in the hospital and usual care at home Control: Assessment of ADL and cognition and usual care in the hospital and at home	**FCT: Barthel Index, Lawton IADL scale** HU: Discharge destination, LOS	Admission Discharge	Between-group differences at discharge: - Barthel Index, Lawton IADL scale: n.s. Feasibility: - Barthel Index, Lawton IADL scale: mean discharge scores in both groups within the best 15% of the rating scale → ceiling effect - Missing data: Barthel Index, Lawton IADL scale = 0% (discharge)
Oldmeadow 2006 [43] Australia	*n* = 60 Mean age: 79 yrs. Females: *n* = 43 (68%) Patient with hip fracture	Intervention: - First walk at day 1 or 2 (early mobilization) (7 days/week) Control: Usual care (first walk at day 3 or 4) (7 days/week)	**MOB: mILOAS items: Transfer from supine to sitting, transfer from sitting to standing (independent vs. assisted), ambulation (walking distance), step negotiation (independent vs. failed/unable)** HU: Discharge destination, LOS	Day 7	Between-group differences at post-surgery day 7: - mILOAS: transfer item: ↑, walking distance: ↑, step negotiation: n.s. Feasibility at day 7: - mILOAS step negotiation item: > 15% (23%) of total sample with worst possible score → floor effect, 21% missing data - mILOAS transfer item = 15% missing data
Siebens et al., 2000 [44] USA	*n* = 300 Mean age: 78 yrs. Females: *n* = 182 (61%); Patients with acute medical illness	Intervention: - Hospital-based exercise program (twice a day) - Flexibility and strengthening exercises - Walking program (60 to 80% max. Heart rate, 5 min to 30 min) Control: Usual care	**HU: LOS** ACE: Mortality	Discharge	Between-group differences at discharge: - LOS: n.s. Feasibility: - Missing data: LOS = 0%
Torres-Sanchez 2017 [45] Spain	*n* = 58 Mean age: 74 yrs. Females: *n* = 16 (28%); Patients with acute exacerbation of COPD	Intervention: - Additional individually-adapted endurance training on a pedal exerciser - Cycling time, velocity, and resistance were adapted to patient and increased every day Control: Usual care (no supervised or progressive exercise)	**MOB: Lower limb strength (handheld dynamometer), balance (OLS), exercise capacity (30CST)**, physical activity/number of steps (SenseWear Armband)	Admission Discharge	Group × time interaction: - Lower-limb strength: ↑ - Balance: ↑ - Exercise capacity: ↑ Between-group differences at discharge: - Lower-limb strength: ↑ - Balance (OLS): ↑ - Exercise capacity (30STS): n.s. Feasibility: - Missing data: Lower-limb strength, balance (OLS), exercise capacity (30CST) = 0%
Asplund 2000 [46] Sweden	*n* = 444 Mean age: 81 yrs. Females: *n* = 251 (61%) Patients with acute medical illness	Intervention: - Multidisciplinary teamwork (internist, geriatrician, nurses, nurse aids, physiotherapist, occupational therapist, social worker, dietician) - Assessment by physiotherapist and occupational therapist - Early start of rehabilitation - Discharge planning Control: General medical unit care	**ACE: Mortality** HU: LOS, discharge destination, hospital costs	Admission Discharge	Between-group differences at discharge: - Mortality: n.s. Feasibility: - Missing data: mortality = 3% 97% survivals → mortality = rare event
Barnes 2012 [47] USA	*n* = 1632 Mean age: 81 yrs. Females: *n* = 1094 (67%) Patient with acute medical illness	Intervention: - Prepared environment (e.g., carpeting, handrails, uncluttered hallways) - Patient-centered care (daily assessment by nurse of physical, cognitive and psychosocial function - Protocols to improve of ADL (bathing/dressing, mobility/transferring, toileting, feeding), nutrition, skin care, falls, cognition, mood etc., daily team rounds by physiotherapist, nurse, social worker, nutritionist) - Planning for discharge - Medical care review (daily by medical director) - Protocols to minimize adverse effects (e.g., urinary catheterization) Control: Usual care	**HU: LOS, hospital costs,** process-of-care measures (physical therapy consults, orders for bed rest, use of physical restraints, documentation of discharge planning, discharge destination) FCT: Katz ADL Index (bathing, dressing, toileting, transferring, eating), Lawton IADL scale (shopping, cooking, performing household chores, using transportation, managing money, managing medication, and using the telephone) MOB: 5-items hierarchical mobility scale ACE: Mortality	Admission Discharge	Between-group differences at discharge: - LOS: ↓ - Hospital costs: ↓ - Feasibility: - Missing data: LOS, hospital costs = NA
Counsell 2000 [48] USA	*n* = 1531 Mean age: 80 yrs. Females: *n* = 926 (61%) Patients with acute medical illness	Intervention: - Prepared environment (e.g., carpeting, handrails, uncluttered hallways) - Patient-centered care (daily assessment by nurse of physical, cognitive and psychosocial function - Protocols to improve of ADL (bathing/dressing, mobility/transferring, toileting, feeding) nutrition, skin care, falls, cognition, mood etc., daily team rounds by physiotherapist, nurse, social worker, nutritionist) - Planning for discharge - Medical care review (daily by medical director) - Protocols to minimize adverse effects Control: Usual care	**FCT: Modified Katz ADL Index** (bathing, dressing, toileting, transferring, eating), modified Lawton IADL scale (shopping, cooking, performing household chores, using transportation, managing money, managing medication, and using the telephone) MOB: PPME, 5-items hierarchical mobility scale HU: Process-of-care measures (nursing care plans, time from admission to initiation of discharge planning, social work consultation, orders for bed rest, physical therapy consults, use of urinary catheters, and application of physical restraints, inappropriate medications), LOS, hospital costs, discharge destination PSY: Caregiver satisfaction ACE: Mortality	Admission Discharge	Between-group differences at discharge: - Mortality: n.s.; Modified Katz ADL Index: n.s. Feasibility: - Missing data: Katz ADL Index = NA (admission & discharge)
Huusko 2000 [49] Finland	*n* = 260 Mean age: 80 yrs. Females: *n* = 184 (72%) Patients with hip fracture No dementia (MMSE 24–30): *n* = 99 (41%) Suspected severe dementia (MMSE 0–11): *n* = 28 (12%) Suspected moderate dementia (MMSE 12–17): *n* = 36 (15%) Suspected mild dementia (MMSE 18–23): *n* = 77 (32%)	Intervention: - Multidisciplinary teamwork (geriatrician, general practitioner, nurses, social worker, neuropsychologist, occupational therapist, physiotherapist) - Geriatric team assessment - Physiotherapy (2times/day), ADL training by nurses - Weekly meetings by physiotherapists and nurses - Discharge plan Control: Discharged to local hospitals	**HU: LOS**	Discharge	Between-group differences at discharge: - LOS: severe dementia (MMSE score: 0–11 pt): n.s.; moderate dementia (MMSE score: 12–17 pt): ↓; mild dementia (MMSE score: 18–23 pt) ↓; normal (MMSE score: 24–30): n.s. Feasibility: - Missing data: LOS = 0%
Landefeld 1995 [50] USA	*n* = 651 Mean age: 80 yrs. Females: *n* = 435 (67%) Patients with acute medical illness	Intervention: - Prepared environment (e.g., carpeting, handrails, uncluttered hallways) - Patient-centered care (daily assessment by nurse of physical, cognitive and psychosocial function - Protocols to improve of ADL (bathing/dressing, mobility/transferring, toileting, feeding) nutrition, skin care, falls, cognition, mood etc., daily team rounds by physiotherapist, nurse, social worker, nutritionist) - Planning for discharge - Medical care review (daily by medical director) - Protocols to minimize adverse effects (e.g., urinary catheterization) Control: Usual care	**FCT: Modified Katz ADL Index** (bathing, dressing, toileting, transferring, eating), Lawton IADL scale MOB: Walking ability HU: Discharge destination, LOS, hospital costs PSY: GDS, overall health status COG: MMSE	Admission Discharge	Between-group differences at discharge: - Katz ADL Index: ↑ Multivariable regression analyses controlled for confounding baseline patient characteristics: IG assignment = significant independent predictor of an increase in the number of independently performed ADLs Feasibility: - Katz ADL Index: > 15% of participants reaching a score within the best 15% of the instrument’s rating scale → ceiling effect - Missing data: Katz ADL Index: 0% (admission & discharge)
Naglie 2002 [51] Canada	*n* = 279 Mean age 84 yrs. Females *n* = 223 (80%); Patients with hip fracture	Intervention: - Multidisciplinary teamwork (physiotherapist, occupational therapist, nurse, social worker) - Special education of staff - Prevention of complications (e.g., delirium, urinary problems, malnutrition) - Physiotherapy: early full weight bearing, ADL training, (2 times/day for 5 day/week) - Discharge plan, pre-discharge home visits - 2 times/week meeting for monitoring treatment plan Control: Usual care	**HU: Discharge destination,** LOS	Admission Discharge	Between-group differences at discharge: - Discharge destination: ↑ (in community-dwellers, relative’s/retirement home residents), n.s. (in nursing home residents) Feasibility: - Missing data: Discharge destination: 0%
Pitkälä 2008 [52] Finland	*n* = 174 Mean age: 83 yrs. Females: *n* = 128 (74%) Patients with delirium	Intervention: - Comprehensive geriatric assessment (physical examination, cognition, nutrition, screening of depression, review of medication) - Administering antipsychotics for hyperactive/psychotic symptoms - Cholinesterase inhibitors - Orientation (calendars, clocks) - Physiotherapy - Nutritional supplements - Comprehensive discharge planning (e.g., occupational home visits) Control: Usual care	**PSY: 15D HRQOL questionnaire, self-developed subjective health scale**	Admission Discharge	Between-group differences at discharge: - HRQOL: ↑ - Self-developed subjective health sale: ↑ Feasibility: - Missing data: 15D questionnaire: 9%; self-developed subjective health sale: NA (admission & discharge)
Prestmo 2015 [30] Taraldsen 2014 [31] Norway	*n* = 397 Mean age: 83 yrs. Females: *n* = 293 (73%) Patients with hip fracture	Intervention: - Multidisciplinary teamwork (geriatricians, nurses, physiotherapists, occupational therapists, with special competence in geriatrics) - Comprehensive geriatric assessment (somatic and mental health, function, social situation) - Interdisciplinary team meetings - Adequate nutrition, - Individual rehabilitation plan based on cognition and motivation - Early mobilization, functioning in ADL, weight-bearing exercise program - Early discharge planning Control: Usual care (standard orthopedic care)	**MOB: SPPB**, **PA (activPAL: time spent in upright**, number of upright events), Cumulated Ambulation Score HU: LOS, discharge destination, hospital costs	Day 4 after surgery (activePAL) Day 5 after surgery (SPPB)	Between-group differences at day 4 (activePAL) and 5 (SPPB): - SPPB: ↑ - Time spent in upright: ↑ Feasibility: - Missing data: SPPB = 13% (5 days after surgery) - activPAL: > 15% missing data
Siebens et al., 2000 [44] USA	*n* = 300 Mean age: 78 yrs. Females: *n* = 182 (61%); Patients with acute medical illness	Intervention: - Hospital-based exercise program (twice a day) - Flexibility and strengthening exercises - Walking program (60 to 80% max. Heart rate, 5 min to 30 min) Control: Usual care	**HU: LOS** ACE: Mortality	Discharge	Between-group differences at discharge: - LOS: n.s. Feasibility: - Missing data: LOS = 0%
Stenvall 2007a,b, 2012 [27–29] Lundström 2007 [26] Sweden	Total sample: *n* = 199 Mean age: 82 yrs. Females: *n* = 148 (74%) Patients with hip fracture	Intervention: - Multidisciplinary teamwork (nurses, physiotherapists, occupational therapists, dietician, geriatrician) - Staff education in prevention of postoperative complication - Individual care planning (all team members assessed each patient as soon as possible, planning of process and goals twice a week) - Prevention and treatment of complications (falls, delirium etc.) - Pain treatment (contained assessment of underlying causes) - Saturation (oxygen-enriched air during first two postoperative days) - Nutrition (protein-enriched meals during the first four days) - Mobilization: (ADL training with focus on fall risk factors, high-intensity weight-bearing exercises) Control: Usual care (no corresponding team work)	**ACE: Falls, fallers,** and time lapse to first fall after admission; **AIS,** postoperative complications (urinary tract infections, decubitus ulcer, sleeping disturbances, mortality) **MOB: COVS walking item** **FCT: ADL staircase** (Katz ADL Index with IADL items) **HU: Discharge destination,** LOS **COG: Number of delirious days (OBS scale), MMSE** **PSY: GDS** BPN: Nutritional problems assessed by care/nursing staff	Discharge	Between-group differences at discharge: - Falls: ↓ - Fallers: ↓ - AIS: minor or moderate injuries:↓, serious injuries: n.s. - COVS walking item: n.s. - ADL staircase: NA (Katz ADL Index: n.s., IADL: NA) - Discharge destination: n.s. - Number of delirious days: ↓ - MMSE: n.s. - GDS: n.s. Feasibility: - Falls: 81% = non-fallers → rare event - AIS: not assessable in 81%; 42% of fallers with an AIS score of 0 pt. → floor effect - GDS: missing data at discharge in 20% - ADL staircase: > 15% of patients reaching a score within the best 15% of the best possible score → ceiling effect
	Subsample: *n* = 64 (32%) Mean age: 82 yrs. Females: *n* = 47 (73%) Patients with hip fracture & dementia Mean MMSE score: 8.6 (IG), 6.9 (CG)		**ACE: Postoperative complications** (pneumonia, urinary tract infection, decubital ulcers, new fracture, falls, fallers, fall incidence rate, mortality) **COG: Number of delirious days (OBS scale)** BPN: Nutritional problems assessed by care/nursing staff **MOB: COVS walking item** **FCT: ADL staircase** (Katz ADL Index with IADL items)		Between-group differences at discharge: - Postoperative complications: total: NA; urinary tract infection: ↓; fallers: ↓; Fall incidence rate: ↓; mortality, pneumonia, decubital ulcers, new fracture: n.s. - Number of delirious days: ↓ - COVS walking item: n.s. - ADL staircase: NA (Katz ADL Index: n.s., IADL: NA)
Vidan 2005 [53] Spain	*n* = 319 Mean age: 82 yrs. Females: *n* = 260 (82%) Patients with hip fracture	Intervention: - Multidisciplinary teamwork (geriatrician, rehabilitation specialist, and specific social worker) - Geriatric assessment (medical, psychosocial problems and functional capability) - Interdisciplinary meeting to elaborate a comprehensive therapeutic plan (weekly repeated) - Daily visits by geriatrician - Rehabilitation specialist planned physiotherapy (schedule, intensity and duration) - Social worker assessed the social environment Control: Usual care	**HU: LOS** **ACE: Mortality, postoperative complications** COG*:* CAM	Admission Discharge	Admission to discharge: - LOS: n.s. - Mortality: ↓ - Postoperative complications: ↓ Feasibility: - LOS: 0% (admission to discharge) - Mortality: 97% survivals → rare event - Postoperative complications: 47% of patients without complications (admission to discharge) → rare events

*10MWT* 10-Meter Walking Test, *30CST* 30-Seconds Chair Stand Test, *6MWT* 6-Minute Walk Test, *ACE* Adverse clinical events, *ADL* Activities of daily living; AIS, Abbreviated Injury Scale, *BPN* Body constitution, physiological or nutritional status, *CAM* Confusion Assessment Method, *CAM* Confusion Assessment Method, *CAT* COPD Assessment Test, *CG* Control group, *COG* Cognitive functioning, *COPD* Chronic obstructive pulmonary disease, *COVS* Clinical Outcome Variables Scale, *CRQ-SAS* Chronic Respiratory Questionnaire Self-Administered Standardized, *DSM-IV* Diagnostic and Statistical Manual of Mental Disorders, *FCT* Functional status, *FIM* Functional Independence Measure, *GDS* Geriatric Depression Scale, *HRQOL* Health-related quality of life, *HU* Hospital outcomes, *IADL* Instrumental activities of daily living, *IG* Intervention group, *LOS* Length of stay, *mDRI* modified Disability Rating Index, *mILOAS* Modified Iowa level of Assistance, *mMRC* modified Medical Research Council, *MMSE* Mini-Mental State Examination, *n.s* not significant (*p* > 0.05), *NA* Not available, *OLS* One Leg Stance, *PPAS* Postoperative patient activity scale, *PPME* Physical Performance and Mobility Examination, *PSY* Psychological status, *SPPB* Short Physical Performance Battery, *TUG* Timed Up and Go, *UCLA scale* University of California, Los Angeles Activity scale; ↑, significant increase (*p* ≤ 0.05); ↓, significant decrease (*p* ≤ 0.05)

### Methodological quality

Total PEDro scores ranged from 2 to 8 points, with a mean score of 6.0 ± 1.7 points. High methodological quality and low risk of bias were found for 17 studies (70.8%), with a PEDro score of > 5 points [27, 31, 32, 34, 39–46, 48, 49, 51–53]. Seven studies (29.2%) did not exceed a score of 5 points, indicating a low methodological quality and high risk of bias [33, 35–37, 47, 50, 54]. The detailed quality scores on the PEDro scale for each RCT are provided in Additional file 3: Table S2.

### Study samples

The mean sample size was 357 ± 421 and varied considerably from 15 [36] to 1632 [47] participants, with half of the studies (*n* = 12, 50.0%) recruiting at least 200 participants [30, 32, 39, 42, 44, 46–51, 53]. Participants’ age across studies averaged 80.0 ± 3.4, with a range from 71 [38] to 85 [33] years. Identified studies predominantly included older patients with general medical conditions (*n* = 12, 50.0%) [32–34, 36, 39, 40, 42, 44, 46–48, 50] or acute hip fracture (*n* = 8, 33.3%) [27, 30, 37, 41, 43, 49, 51, 53]. Other patient characteristics for study inclusion were acute exacerbation of chronic obstructive pulmonary disease (COPD) (*n* = 2, 8.3%) [38, 45], delirium (*n* = 1, 4.2%) [52], or abdominal surgery (*n* = 1, 4.2%) [35].

### Interventions

Early inpatient rehabilitation interventions could basically be divided into two categories: (1) “hospital usual care” with an additional or modified exercise program as included in 14 studies [32–45] or (2) multidisciplinary programs with an exercise component as included in 10 studies [27, 30, 46–53]. In the following, we refer to these two categories as exercise interventions and multidisciplinary programs, respectively.

Multidisciplinary intervention teams usually consisted of geriatricians, nurses, physical therapists, occupational therapists, dieticians, and/or social workers. Apart from the exercise component, multidisciplinary programs included components of comprehensive geriatric assessment [27, 30, 46–50, 52, 53], multidisciplinary team meetings and individual care planning [27, 30, 46–51, 53], discharge planning [30, 46–53], nutritional interventions [27, 30, 47, 48, 50, 52], prevention and treatment of complications (e.g., vitamin supplementation, screening of infections) [27, 51], cognitive interventions [26, 47, 48, 50, 52], psychological interventions [47, 48, 50, 52], staff education [27, 51], or specifically-designed environments [47, 48, 50].

The content of the exercise component of the multidisciplinary programs most frequently included ADL training [27, 30, 47–51] and/or strength training [27, 30, 51]. Three studies did not provide detailed information on the content of the exercise component apart from stating that it included physical and/or occupational therapy [46, 52, 53].

Exercise interventions were usually supervised by physiotherapists, occupational therapists, nurses, allied health assistants, or staff specifically trained by physiotherapists. Intervention contents included modified or additional exercises with walking training [36, 40–44], strength training [33, 39–41, 44], ADL training [32, 36, 37, 42], flexibility training [38, 44], lower-limb endurance training [38, 45], cognitive exercises [32, 39], balance training [40], transfer training [40, 41], physical activity (PA) behavior intervention [34, 38], IADL training [36], breathing exercises [38], and/or proprioceptive neuromuscular facilitation exercises [35].

Participants in the control groups of the studies generally received usual care according to the general routines of the hospital they were admitted to.

### Outcome measures

Identified outcome measures varied considerably among the included studies, with a total of 33 different primary outcome measures. They can be grouped into the following eight categories: (1) functional status, which refers to measures of (I) ADL; (2) mobility status, which refers to measures of motor performance or PA behavior; (3) hospital outcomes, which refers to measures of healthcare utilization during hospitalization (e.g., length of stay [LOS], hospital costs]; (4) adverse clinical events, which refer to measures of falls, medical complications, or mortality; (5) psychological status, which refers to measures of health-related quality of life (HRQOL), anxiety, depression, or confidence; (6) cognitive functioning, which refers to measures of global cognitive status or transient cognitive dysfunction (e.g., delirium); (7) body constitution, physiological or nutritional status, which refers to measures of lean and fat tissue mass, body weight, nutritional intake, or biochemical outcomes (e.g., serum albumin); and (8) disease-specific outcomes (e.g., COPD *severity, exacerbation* rates*)*. In the following, the different primary outcome measures used across the included studies were described for each category. Due to their specificity, the disease-specific outcome measures were not further analyzed and discussed in this review.

#### Functional status

Functional status was assessed in 11 studies (45.8%; 8 exercise interventions [32–37, 40, 42] and 3 multidisciplinary programs [28, 48, 50]) using an (I) ADL measure only [32–36, 40], both an ADL and IADL measure [37, 42, 48, 50], or a combined (I) ADL measure [28]. The most frequently used (I) ADL instruments were the Katz ADL Index [33, 34, 48, 50], the Barthel Index [32, 40, 42], and the Lawton IADL scale [35, 42]. Other functional status measures included the Functional Independence Measure (FIM [36]), modified Disability Rating Index (mDRI) and modified Klein-Bell [KB] ADL scale [37], or the ADL staircase (Katz ADL Index extended by further IADL items) [28].

#### Mobility status

Mobility status was assessed in seven studies (29.2%; 5 exercise interventions [35, 38, 41, 43, 45] and 2 multidisciplinary programs [28, 30]). Nine different motor performance measures were identified, including the modified Iowa Level of Assistance Scale (mILOAS) [41, 43], the Timed Up and Go (TUG) [35, 41], the walking item of the Clinical Outcome Variables Scale (COVS) [28, 29], the Short Physical Performance Battery (SPPB) [30], a lower extremity handheld dynamometry strength measurement [45], the One Leg Stance (OLS) and 30-s Chair Stand Test (30CST) [45], the 10-Meter Walking Test (10MWT) [35], the 6-Minute Walk Test (6MWT) [38], and a self-developed postoperative patient activity scale (PPAS) [35]. PA measures were reported in only two studies, including the self-administered University of California, Los Angeles Activity (UCLA) scale [35] or an accelerometer-based PA monitor (activPAL) [31].

#### Hospital outcomes

Hospital outcomes were assessed in six studies (25.0%; 5 multidisciplinary programs [28, 47, 49, 51, 53] and 1 exercise intervention [44]). LOS was reported in all these studies. Further outcome measures included discharge destination [28, 47, 51] or hospital costs and other process-of-care measures (e.g., physical therapy consults, orders for bed rest) [47].

#### Adverse clinical events

Three studies (12.5%; 3 multidisciplinary programs) assessed mortality [46, 53], different complications during hospitalization [53], or falls/fall-related outcomes (Abbreviated Injury Scale [AIS]) [27].

#### Psychological status

Psychological factors were assessed in three studies (12.5%; 2 multidisciplinary programs [26, 52] and 1 exercise intervention [36]), using the Geriatric Depression Scale (GDS) [26], the 15D HRQOL questionnaire [52], or the Self-Efficacy Gauge and Life-Satisfaction Index [36].

#### Cognitive functioning

Two studies (8.3%; 1 exercise intervention [39] and 1 multidisciplinary programs [26]) used the Confusion Assessment Method (CAM) to assess the number of delirious patients [39] or the Organic Brain Syndrome (OBS) scale to screen for the number of delirious days during hospitalization and the Mini-Mental State Examination (MMSE) to screen the global cognitive status [26].

### Matching of outcome measures

Table 3 presents the results of the matching procedure and the intervention effects reported for each outcome measure identified among studies. In the following, the results of the matching procedure were initially summarized for each outcome category.

**Table 3:** Results of the matching procedure and intervention effects reported for each outcome measure

Outcome measures		Study	Matching				Intervention effects
Category	Instrument		Intervention	Sample		Setting	
				Floor/ceiling effects or rare event	Missing data		
FCT	(modified) Katz ADL Index	Blanc-Bisson 2008 [33]	–	+	+	+	NA
		Brown 2016 [34]	±	–	+	+	n.s.
		Counsell 2000 [48]	+	+	+	+	n.s.
		Landefeld 1995 [50]	+	–	+	+	↑
	Barthel Index	Abizanda 2011 [32]	±	+	+	+	n.s.
		Jones 2006 [40]	±	–	+	+	n.s.
		Nikolaus 1999 [42]	±	–	+	+	n.s.
	Lawton IADL scale	Czyzewski 2013 [35]	–	–	–	–	NA
		Nikolaus 1999 [42]	–	+	+	–	n.s.
	ADL staircase	Stenvall 2007, 2012 [28, 29] Lundström 2007 [26, 28]	±	–	–	±	NA
	FIM	Eyres 2005 [36]	±	+	+	+	NA
	mDRI	Hagsten 2004 [37]	±	NA	+	±	n.s.
	mKB ADL scale	Hagsten 2004 [37]	+	+	+	±	↑
MOB	6MWT	He 2015 [38]	+	+	+	+	NA
	10MWT	Czyzewski 2013 [35]	±	+	+	+	NA
	30CST	Torres-Sanchez 2017 [45]	±	+	+	+	↑
	mILOAS						
	total score	Kimmel 2016 [41]	+	+	+	+	n.s.
	ambulation item	Oldmeadow 2006 [43]	+	NA	+	+	↑
	step negotiation item	Oldmeadow 2006 [43]	±	–	–	+	n.s.
	transfer items	Oldmeadow 2006 [43]	±	NA	–	+	↑
	activPAL	Taraldsen 2014 [31]	±	+	–	+	↑
	Handheld dynamometry	Torres-Sanchez 2017 [45]	±	+	+	+	↑
	OLS	Torres-Sanchez 2017 [45]	±	+	+	+	↑
	PPAS	Czyzewski 2013 [35]	±	NA	+	+	↑
	SPPB	Prestmo 2015 [30]	+	+	+	+	↑
	TUG	Czyzewski 2013 [35]	±	+	+	+	NA
	UCLA scale	Czyzewski 2013 [35]	±	NA	–	±	NA
	COVS	Stenvall 2007, 2012 [28, 29] Lundström 2007 [26]	+	NA	+	+	n.s.
HU	LOS	Barnes 2012 [47]	+	+	+	+	↑
		Huusko 2000 [49]	+	+	+	+	↑
		Siebens 2000 [44]	–	+	+	+	n.s.
		Vidan 2005 [53]	±	+	+	+	n.s.
	Discharge destination	Naglie 2002 [51]	+	+	+	+	↑
		Stenvall 2007 [28]	±	+	+	+	n.s.
	Hospital costs	Barnes 2012 [47]	+	+	+	+	↑
ACE	Medical complications	Stenvall 2012 [29]	+	NA	+	+	NA
		Vidan 2005 [53]	+	+	+	+	↑
	Mortality	Asplund 2000 [46]	±	–	+	+	n.s.
		Vidan 2005 [53]	±	–	+	+	↑
	AIS	Stenvall 2007,2012 [27–29]	+	–	–	+	↑
	Falls	Stenvall 2007 [27]	+	+	+	+	↑
PSY	Self-Efficacy Gauge	Eyres 2005 [36]	±	+	+	+	NA
	Life Satisfaction Index	Eyres 2005 [36]	–	+	+	+	NA
	GDS	Lundström [26]	–	+	–	+	n.s
	15D HRQOL	Pitkälä 2008 [52]	±	+	+	+	↑
COG	CAM	Jeffs 2013	±	–	+	+	n.s
	OBS scale	Lundström 2007 [26]	+	–	+	+	↑
	MMSE	Lundström 2007 [26]	±	+	+	+	n.s.

*6MWT* 6-Minute Walk Test, *10MWT* 10-Meter Walking Test, *30CST* 30-Seconds Chair Stand Test, *AIS* Abbreviated Injury Scale, *CAM* Confusion Assessment Method, *COVS* Clinical Outcome Variables Scale, *FIM* Functional Independent Measure, *GDS* Geriatric Depression Scale, *HRQOL* Health-related Quality of Life, *LOS* Length of stay, *mDRI* modified Disability Rating Index, *mILOAS* modified *Iowa* Level of Assistance Scale, *mKB ADL scale* modified Klein-Bell ADL scale, *MMSE* Mini-Mental State Examination, *OBS scale* Organic Brain Syndrome scale, *OLS* One Leg Stance, *PPAS* Postoperative Patient Activity Scale, *SPPB* Short Physical Performance Battery, *TUG* Timed Up and Go, *UCLA scale* University of California, Los Angeles Activity scale

+, “match”; ±, “limited match”; −, “no match”; NA, not available; ↑, significant between-group differences in favor of the intervention group (*p* ≤ 0.05); n.s., no significant between-group differences in favor of the intervention group (*p* > 0.05)

#### Functional status

Most frequently, functional measures matched the intervention contents only to a limited extent with items not part of the functional intervention component (e.g., Katz ADL Index ➔ only basic transfer and ambulation training) [28, 32, 34, 36, 37, 40, 42]. Functional measures that specifically addressed the functional intervention contents (e.g., Katz ADL Index → ADL training to improve bathing/dressing, mobility/transferring, toileting, feeding) were used in only three studies [37, 48, 50]. In another three studies, we identified functional measures that did not directly match the interventions, which did not include a functional training component (e.g., Lawton IADL scale → no IADL training content) [33, 35, 42].

Six studies suggested ceiling effects for at least one of their functional measures, with > 15% of participants reaching a score within the best 15% of the rating scales (Katz ADL Index [50], Barthel Index [40], ADL staircase [28]), or mean scores of the sample within the best 15% of the rating scale (Barthel Index [42], Katz ADL Index [34], Lawton IADL scale [35]). A missing data rate of ≥15% for functional measures were reported in two studies, which did not present any data for the Lawton IADL scale [35] or incomplete data for the ADL staircase (only ADL items presented) [28] at discharge.

Two studies used the Lawton IADL scale [35, 42], which did not match to the acute care hospital setting with inappropriate items addressing instrumental home or community activities such as washing, housekeeping, or shopping. Two studies used functional measures (mDRI [37], ADL staircase [28]) that matched to the acute care hospital setting only to a limited extent, including both setting-specific basic ADL items but also setting non-specific IADL items.

#### Mobility status

Most frequently, mobility measures specifically matched the mobility intervention component (e.g., 6MWT → lower limb endurance training) [28, 30, 38, 41, 43]. Limited matches in which the mobility measure covered the mobility intervention component only to a limited extent (e.g., OLS → chair-based pedal exercises; mILOAS transfer, step negotiation and ambulation items → only walking training) were found in four studies [31, 35, 43, 45].

Only one study suggested a floor effect, with almost one fourth (23.3%) of the total sample reaching a score within the worst 15% of rating scale of the mILOAS step negotiation item [43]. A missing data rate of ≥15% for mobility measures were reported in three studies [31, 35, 43]. Two of them did not present any or incomplete data for the UCLA (missing data: 100%) [35] or single mILOAS items (missing data: 15% [transfers]; 21% [step negotiation] [43]). The other study reported that in 19% of the sample, sensor-based PA data were missing due to reasons such as sensor removing, technical problems, or medical reasons [31].

Most studies used mobility measures specifically addressing mobility or physical activities that can be appropriately assessed within the acute care hospital setting (e.g., SPPB ➔ functional mobility; 10MWT → walking) [28, 30, 38, 41, 43, 45].

Only one study used the UCLA to assess PA behavior, which matched to the acute care hospital setting only to a limited extent, with inappropriate response items addressing intensive physical activities (e.g., swimming, bicycling) or impact sports [35] rather than rehabilitation-specific activities.

#### Hospital outcomes

Three studies used hospital outcomes (LOS, hospital costs, discharge destination) that specifically addressed their intervention components [47, 49, 51]. All these studies conducted a multidisciplinary program that included multidisciplinary team meetings with individual care planning, comprehensive geriatric assessments, and/or discharge planning. Limited matches were found for two other multidisciplinary intervention studies which assessed LOS [53] or discharge destination [28]; however, without including specific discharge planning procedures within their multidisciplinary program. No match was found for one study, which was the only one that assessed the unspecific effect of an additional exercise intervention on a hospital outcome (LOS) [44].

Ceiling and floor effects or rare events were not apparent for any of these setting-specific hospital outcomes, and none of the six studies reported missing data.

#### Adverse clinical events

Two studies analyzing adverse clinical events used outcome measures that specifically matched to the intervention. Both of them assessed the incidence of medical complications during hospitalization to evaluate the specific effect of their intervention contents focusing on the identification, prevention and treatment of these complications [29, 53]. One of these studies also assessed the effect of a systematic assessment and treatment of fall risk factors by the number of falls/fallers and the AIS that specifically matched to this specific intervention component [27, 29]. Two studies assessed mortality during hospitalization, which were addressed to a limited extent by the increased, multidisciplinary diagnostic progress, the improved therapeutic care planning, and the increased patient contact time during acute hospitalization [46, 53].

In both studies assessing mortality, a mortality rate of only 3% during hospitalization was observed [46, 53], indicating a rare event. The AIS used to assess fall-related injury severity showed a ceiling effect with 42% of fallers reaching the best possible AIS score and missing data for 81% of participants who had not fallen [27]. For medical complications, falls, and mortality, no missing data were reported in all studies [27, 46, 53].

Adverse clinical events were appropriately assessed based on nursing/medical records or patient charts in all studies [27, 29, 46, 53].

#### Psychological status

None of the studies focusing on psychological status used a psychological measure that specifically matched their intervention contents [26, 36, 52]. Limited matches were found in two studies, using the 15D HRQOL with single items that were addressed by the intervention contents (15D HRQOL mobility dimension → physiotherapy, 15D HRQOL mental function dimension → orientation training) [52] or the Self-Efficacy Gauge, which has been specifically developed to assess self-perceived confidence in occupational performances, to evaluate an additional occupational therapy program [36]. Psychological measures (Life-Satisfaction Index [36], GDS [26]) that did not match a specific content of their interventions were found in two studies.

Ceiling or floor effects were not identified for any psychological measure [26, 36, 52], and only one study reported a missing data rate of 20% for the GDS at discharge [26].

All psychological measures used in the studies addressed constructs that can be appropriately assessed within the acute care hospital setting.

#### Cognitive functioning

In one of the two studies analyzing cognitive functioning, the number of delirious days as assessed by the OBS scale specifically matched the intervention contents of active preventing, detecting, and treating delirium [26]. The same study also used the MMSE, which matched this intervention component only to a limited extent not including any further cognitive training contents [26]. In the other study, the CAM also only to a limited extent matched in evaluating the effect of additional orientation exercises on the number of delirious patients [39].

For the number of delirious days, a ceiling effect was identified, with 65% of patients having no delirious day [26], and the number of delirious patients represented a rare event, with only 5.4% of patients having a delirium episode during hospitalization [39].

All cognitive measures could be rated as appropriate for use in the acute care hospital setting.

### Intervention effects in relation to the matches

In the following, the main findings reported on the primary outcomes were related to the results of the matching procedure. Details on the intervention effects on the outcome measures identified among studies can be found in Table 3.

#### Functional status

Seven studies (4 exercise interventions [32, 34, 37, 40, 42] and 2 multidisciplinary programs [48, 50]) reported on between-group differences in functional status at hospital discharge, whereas four studies (3 exercise interventions [33, 35, 36] and one multidisciplinary programs [28]) did not. In those studies (*n* = 5) with no or only limited matches between functional measures and exercise intervention, no significant benefits of the intervention could be documented [32, 34, 37, 40, 42]. Only in those two studies where the functional measures specifically addressed the exercise intervention [37], or an intervention component of the multidisciplinary program [50], a significant superior effect of the intervention on the functional status was identified.

#### Mobility status

Six studies (5 multidisciplinary programs [28, 47, 49, 51, 53] and 1 exercise intervention [44]) reported on between-group differences in mobility status after surgery or at hospital discharge based on a variety of 11 different mobility measures. One study only analyzed within-group changes for the mobility outcomes at hospital discharge [38].

Out of the four mobility measures with intervention-specific matches, two (SPPB, mILOAS ambulation item) revealed a significant benefit of the additional exercise intervention [43] or the multidisciplinary program [30] over the usual care on motor performance, whereas the other two did not (COVS walking item [28], mILOAS [41]). All other seven mobility measures with limited intervention-related matches (handheld dynamometry, OLS, 30CST, mILOAS step negotiation and transfer items, PPAS, activPAL) revealed significant beneficial effects in the experimental groups (3 exercise interventions [35, 43, 45] and 1 multidisciplinary program [31]), except for one (mILOAS step negotiation) [43].

Out of the mobility measures that did not reveal significant between-group differences, two covered single subjective rating items of more comprehensive assessment scales (COVS walking item, mILOAS step negotiation item) [28, 43], with partly floor effects in the sample (mILOAS step negotiation item) [43], and one was a comprehensive assessment scale combining subjective rating and objectively-measured items (mILOAS total score) [41].

#### Hospital outcomes

Six studies (5 multidisciplinary programs [28, 47, 49, 51, 53] and 1 exercise intervention [44]) analyzed between-group differences in LOS, discharge destination, and/or hospital costs at hospital discharge. Significantly shorter LOS, more patients reintegrated into the community, and lower hospital costs among the intervention group were found only for these three studies in which the hospital outcomes specifically matched the intervention components of the multidisciplinary programs [47, 49, 51]. No significant between-group differences could be documented [28] in multidisciplinary studies with only limited matches between the hospital outcomes (LOS, discharge destination) and their intervention components [28] and in the exercise intervention study showing no match [44].

#### Adverse clinical events

Between-group differences in adverse clinical events at hospital discharge were analyzed in three multidisciplinary intervention studies [29, 46, 53]. Two studies assessing adverse clinical events that specifically matched their intervention components reported a significant lower number of falls, fallers and minor to moderate fall-related injuries [27] and reduced medical complications in favor of the intervention [53]. Out of the two studies that analyzed (also) mortality, which matched as an outcome measures only to a limited extent to the multidisciplinary interventions during early inpatient rehabilitation in the acute care hospital setting, one reported a significant effect of their intervention in reducing mortality during hospitalization [53], whereas the other study did not [46].

#### Psychological status

Two multidisciplinary studies analyzed between-group differences in HRQOL [52] and/or depression [26] at hospital discharge. In these two studies, a significant psychological benefit of the intervention compared to usual care was observed only by using the 15D HRQOL that showed a limited match, with single dimensions specifically addressing an intervention component [26, 52]. The GDS, as used in one of these studies, did not match the intervention and revealed no significant between-group differences [26].

#### Cognitive functioning

Two studies (1 multidisciplinary program [26] and 1 exercise intervention [39]) analyzed between-group differences in cognitive functioning during hospitalization. For the cognitive measures with limited matches to the intervention (CAM [delirious patients], MMSE), both studies reported no significant benefit of the intervention compared to the usual care [26, 39]. Only for the number of delirious days as assessed by the OBS scale, which specifically matched the intervention component of active prevention, detection and treatment of delirium within the multidisciplinary program, significant between-group differences in favor of the intervention group were reported [26].

## Discussion

The aim of this review was to analyze the matching of outcome measures used in previous RCTs on early rehabilitation in acutely hospitalized older patients to the specific study characteristics (intervention, sample, and setting) and to evaluate the effects of matching on the main findings reported in these RCTs. In the 24 studies included in this review, the selection of primary outcome measures differed considerably, with a total of 33 different outcome measures across six different outcome categories. The matching process indicated also a large heterogeneity in the appropriateness of the selected outcome measures for the intervention contents, the study sample, and the acute geriatric hospital setting. Our findings suggest that a good match especially between the outcome measure and the intervention contents seems to have increased the likelihood for documenting significant intervention-induced benefits among the included studies.

### Functional status

Functional status defined as (I) ADL functioning has become a key outcome during hospitalization in older patients [55]. The ability to perform (I) ADL is a crucial part of human functioning, disability and health, as located centrally in the model of the International Classification of Functioning, Disability and Health (ICF) from the World Health Organization [56], and a major established outcome for rehabilitation. It was therefore not surprising that the primary outcome measures most frequently used in the included studies focused on (I)ADL. However, there was a large heterogeneity in assessing (I) ADL functioning, with seven different (I) ADL instruments identified among the studies. Our findings supports the lack of consensus regarding measuring the functional status of acutely hospitalized older patients in clinical research, as previously reported in a systematic review on the variability of (I) ADL measures in this patient population [57].

Most frequently, the various functional measures addressed ADL rather than IADL. This might be related to the fact that ADL measures assess basic activities essential for an individual’s direct self-care (e.g., bathing, dressing, walking) which are primarily targeted by treatments during the early rehabilitation phase in the acute care hospital setting. In contrast, IADL measures assess more complex activities that are not necessarily a precondition for basic functions, but that are more concerned with self-reliant functioning in the home (e.g., food preparation, housekeeping) or community environment (e.g., shopping, transportation), being rather addressed in the later rehabilitation phases or after hospital discharge. None of the studies using an IADL measure specifically targeted such home or community activities by their intervention [35, 42]. Based on these mismatches of IADL measures with the acute care hospital setting and the intervention contents, none of these studies reported favorable IADL outcomes for their intervention groups [35, 42]. The majority of the studies with a primary IADL or a combined (I) ADL measure even did not present any data for the IADL measures [35] or analyzed only ADL items but not IADL items of the combined (I) ADL measure at hospital [28], which might suggest that IADL functioning was not assessed, potentially also due to the mismatch of measuring IADL in the acute care hospital setting, as discussed before.

For studies using ADL measures, we predominantly found only limited matches between these instruments and the intervention contents [28, 32, 34, 36, 37, 40, 42]. None of these studies revealed a beneficial intervention effect on the functional status. This might be related to the fact that their interventions specifically addressed only a limited number of ADL items such as transferring, walking, or bathing; while other items (e.g., bowel and bladder control), which show limited responsiveness to available interventions, were not addressed. Even if a beneficial effect on addressed items occurred, the impact on ADL instrument’s overall scores, as analyzed in all these studies, might have been too small to reveal significant benefits related to the intervention.

The only two studies reporting better ADL functioning in their intervention groups at discharge used modified ADL instruments, excluding the items that were not contents of their interventions (e.g. eating, incontinence) [37, 50]. Such modifications may increase the specificity and sensitivity of the outcome measure and, in turn, seem to increase the probability to capture significant intervention effects, as suggested by the significant findings of the two studies. However, it must be kept in mind that modified assessment instruments are no longer validated, thus requiring further psychometric testing before their application [16].

Another potential explanation for insufficient intervention effects on (I) ADL functioning might be related to the ceiling effects identified for most of the ADL instruments already at hospital admission (Barthel Index [40], (modified) Katz ADL Index [34, 50], ADL staircase [28], Lawton IADL scale [35]), indicating a mismatch between these instruments and the characteristics of the sample. If patients’ scores are close to the top of the scale (i.e. at the ceiling) already at baseline, there is only little room for further subsequent improvements, substantially reducing an instrument’s sensitivity as well as a study’s ability to detect significant changes in those patients [14, 58]. As already recommended previously [8], future studies may therefore use functional measures that cover a broader range of ability levels for acutely hospitalized older patients to explore the effects of early rehabilitation in this highly heterogeneous patient population.

### Mobility status

Mobility is fundamental to healthy aging and quality of life in older adults [59], and a loss of mobility can result in a decline in autonomy [60]. Consequently, measuring mobility can determine the level of independence and the health care needs in the older population [61]. Measures addressing the patients’ mobility status formed the second largest category of primary outcome measures. Surprisingly, we identified an even greater heterogeneity of instruments on mobility status than reported above for functional status. None of the primary mobility instruments was used in more than one study, except for the mILOAS. However, also the mILOAS was used differently in two studies, analyzing either the total score [41] or only individual items (walking, step negotiation, transfers) [43]. Our findings on this heterogeneity are in line with a previous systematic review on instruments used to evaluate mobility of older patients during hospitalization [62], highlighting that the lack of consensus not only includes functional but also mobility measure in this setting.

For none of the mobility measures, we identified a total mismatch with a study’s intervention contents, probably based on the fact that this review considered only studies which included a physical exercise intervention [32–45] or a multidisciplinary program with physical exercise as a training component [27, 30, 46–53]. Even if the specific physical intervention content was not directly matched by most of the mobility measures – for example, in terms of conducting physical exercise on specific motor abilities (e.g., pedal exercise → endurance) but assessing other motor abilities (e.g., OLS → balance) – both the mobility measure and the intervention content were related to the overarching construct of mobility, leading to at least limited matches between those. Most frequently, these mobility measures with limited intervention-specific matches still revealed significant effects in favor of the intervention groups compared to the usual care groups. This finding suggests that mobility measures seem to be more sensitive to detect potential intervention-induced effects than the functional measures discussed above, for which a rather high specificity (“perfect match”) to the intervention content was required to reveal such significant between-group differences.

Another advantage of the mobility measures and rationale for their higher potential to detect intervention-induced changes compared to the functional measures might be seen in their coverage of a broader spectrum of patients’ abilities in the highly heterogeneous population of older patients. We identified no ceiling or floor effects for primary mobility measures, except for one study reporting a floor effect for a single item of the mILOAS (negotiation item) [43]. However, no floor effects occurred when its total score was used, as reported in another study [41].

Considering the instrument format of the mobility measures used in the studies analyzing between-group differences (i.e. subjective, observation-based or more standardized, objective measurement methods), it is conspicuous that those measures which did not reveal intervention effects were based on subjective, observation-based rating items (COVS walking item [28], mILOAS step negotiation item [43]) or a more comprehensive assessment scale including predominantly subjective items (mILOAS) [41]. In contrast, all objective mobility measures, for which between-group differences were analyzed (SPPB, handheld dynamometry, OLS, 30CST, mILOAS ambulation item [walking distance], activPAL), revealed favorable mobility outcomes for the intervention group [30, 43, 45], suggesting that this instrument format seems to be more sensitive to show the benefit of exercise-based interventions.

The mobility measures most frequently used addressed key motor functions such as standing, walking, and/or transferring (e.g., SPPB, 10MWT, 30CST, TUG) [30, 35, 45], which are crucial for functional mobility and independence in daily life [62, 63]. PA behavior as a more complex, multidimensional construct was primarily investigated in only 2 studies (UCLA [35], activPAL [31]), with only one of them presenting PA data at discharge [31]. This study revealed a positive intervention effect on PA behavior assessed by a sensor-based PA monitor. Using such highly objective PA assessment instruments might be a promising approach to demonstrate intervention-induced effects; however, it might also be associated with feasibility issues in the sample of older patients, as a high missing data rate was reported in this study (19%). As indicated in a previous review on the utility and accuracy of PA sensors in older hospitalized patients, further research is required to examine their feasibility as well as their validity in this patient population [64].

### Hospital outcomes

LOS, hospital costs, or discharge destination are outcomes associated with healthcare utilization or medical service use in a broad sense and are related to a series of potential cost-saving factors for healthcare [65]. For example, a reduction of LOS can decrease inpatient hospital costs and increase hospital bed availability, increasing the overall cost-efficiency of hospitals [66]. Given the great importance of such cost-related outcomes, it was not surprising that they were the third largest category of primary outcomes identified in this review. LOS was the most frequently evaluated hospital outcome, which might be related to the fact that this hospital outcome may be considered as the key driver of inpatient costs [38] and as an indicator of hospital efficiency [67].

Within our matching procedure, it was initially assumed that changes in hospital outcomes require an optimized organizational proceeding between different in-hospital disciplines, i.e. a multidisciplinary intervention program. This assumption was based on previous findings made by de Morton (2007), suggesting that improvements in these outcomes might result from a better coordination of care provision, increased medical, nursing or allied health interventions, a combination of improved team goal setting and discharge planning, and/or increased patient contact time during acute hospitalization [8]. Therefore, matches or limited matches between hospital outcomes and intervention contents were given only for multidisciplinary studies. Among these multidisciplinary studies, however, only those with intervention contents strictly optimized to the hospital outcome (e.g., discharge destination → discharge planning) revealed significant intervention-induced benefits [47, 49, 51]. All other multidisciplinary studies that used hospital outcomes with only limited matches to the intervention contents (e.g., discharge destination → only individual care planning but no specific discharge planning) could not document such beneficial effects [28, 53]. The only study evaluating an exercise-only intervention by using LOS as a primary outcome [44], which resulted in a mismatch with the intervention contents, was unable to detect significant between-group differences. Hospital outcomes seem not to be sufficiently specific and sensitive enough to document unspecific effects of an exercise intervention and may therefore not be considered as the first choice for the evaluation of interventions with a mere exercise focus in the acute geriatric hospital setting [9]. Our findings support the initial assumption that hospital outcomes might be able to reveal benefits of multidisciplinary programs; however, only if the intervention contents were specifically addressed by the intervention contents.

On the other hand, hospital outcomes are based on a simple data acquisition with high specificity to the hospital setting, as indicated by the overall lack of missing data in all the studies primarily analyzing hospital outcomes [28, 44, 47, 49, 51, 53]. Outcomes such as LOS, hospital costs, or discharge destination are usually based on highly objective, reliable and precise data, which are already captured within the routine hospital records, requiring only little additional effort for data acquisition.

### Adverse clinical events

An adverse clinical event can generally be described as an acute clinical problem that newly occurred during hospitalization and was not present at hospital admission [68]. According to previous systematic reviews on the effects of physical exercise intervention in acutely hospitalized older patients [8, 63], the identified outcome measures such as falls, medical complications, and mortality were categorized as clinical adverse events also in this review. This category of outcome measures stands out as it does not focus on functioning and disability following the established rehabilitation paradigm of the ICF framework [56] but rather focuses on patients’ acute clinical problems and medical conditions. This might also provide a reasonable explanation for the non-frequent use of primary outcome measures out of this category. If adverse clinical events were investigated in the included studies, they were most frequently (6 out of 9 studies) defined as a secondary outcome [34, 40, 41, 44, 47, 48], and only three studies, defined them as a primary outcome [27, 46, 53], with all of them evaluating multidisciplinary program.

More or less, all outcome measures of this category represent rather rare events (e.g., injuries falls, mortality), with the consequence that even in high-risk groups for such outcomes, it may need very large sample sizes and/or highly specific and extraordinary effective intervention strategies to reveal significant improvements over the limited time period of acute care hospitalization. In addition, adverse clinical events can be related to a variety of different factors such as system failures, involuntary errors, or negligence [69]. A multidisciplinary approach was therefore considered to be an essential basic requirement for a match between the outcome category of adverse clinical events and the intervention. In studies analyzing the effects of a multidisciplinary program on medical complications or falls, the intervention contents were indeed strictly optimized to reduce such adverse clinical events (e.g., treatment of fall risk factors → number of falls; identification, prevention and treatment of complications ➔ postoperative complications), leading to significant benefits induced by their multidisciplinary programs compared to usual care [29, 53].

Mortality was used as a primary outcome in two multidisciplinary studies [46, 53]. Reducing mortality is certainly one of the most desirable goals in clinical health care. Mortality can be easily, objectively and reliably measured, as also indicated by lack of missing data among these two studies [46, 53]. However, it can also be described as the “hardest outcome of all”, as mortality rates can be affected by many factors other than the contents or quality of clinical care [70] that cannot all be controlled for in a RCT. Based on the complexity of mortality, only limited matches to the intervention approach with primary focus on functional rehabilitation had been achieved in both studies, even if the multidisciplinary programs included intervention contents that might be beneficial for preventing mortality (e.g., increased patient contact time, multidisciplinary diagnostic progress). The very low mortality rates (< 3%) emphasize the assumption that mortality fortunately represents a rare event, even in the high-risk group of acutely hospitalized older patients. To allow for the documentation of a successful intervention on such rare events, large sample sizes combined with highly effective intervention strategies are required to allow for documentation of a successful intervention. Based on low mortality rates and the limited matches to the interventions, it was surprising that one of them reported a significant between-group difference in favor of their intervention group [53]. However, as also mentioned by the authors of this study, this finding has to be interpreted with caution. Although the relative intervention-induced reduction in mortality seems huge (− 89%), because the absolute number of deaths was low in both groups (control group: *n* = 9 vs. intervention group: *n* = 1), they could not formally exclude that this between-group difference was due to chance.

### Psychological status

The psychological measures used as primary outcomes addressed different psychological constructs such as depression, self-efficacy, life satisfaction, or quality of life. Only three studies defined such measures as a primary outcome, indicating that psychological constructs were not a main focus of the studies identified in this review. None of the interventions of the studies with a primary psychological measure had a clear interventional approach to target psychological factors [26, 36, 52], suggesting that in these studies it was assumed that intervention contents might be indirectly associated with relevant psychological side effects. Out of the 2 studies analyzing between-group differences in psychological outcomes [26, 36, 52], only one study revealed a psychological benefit of the intervention. The fact that this study used a multidimensional psychological measure (15D HRQOL) with dimensions (e.g., mobility, mental function) that addressed some intervention contents at least to a limited extent (e.g., psychotherapy, orientation training) might explain this rather unspecific effect [52]. The other study could not document intervention-induced psychological benefits, which might be a direct consequence of the mismatch between the selected psychological outcome measure (GDS) and the intervention program [26].

### Cognitive functioning

Cognitive functioning also was not a main focus of the identified studies, as only two of them defined global cognitive status (MMSE) and/or delirium (OBS scale, CAM) as a primary outcome [26, 39]. Among these two studies, only the specific multidisciplinary intervention with focus on active prevention, detection and treatment of delirium showed beneficial effects [26]. The same study was, however, not able to document intervention-induced effects on the patients’ global cognitive status, which may be related to the fact that in addition the delirium-related, acute cognitive intervention contents, the multidisciplinary program included no further cognitive intervention contents that specifically addressed cognitive functioning more globally as assessed by the MMSE.

The other study could not document an intervention-induced effect on the number of delirious patients as assessed by the CAM during hospitalization; however, the intervention of this study only included a cognitive intervention content that seemed not specific enough for delirium treatment, in terms of an orientation program [39]. Another potential explanation might be the low incident of delirium in the sample of this study (< 6%), reducing the power to detect a significant intervention effect, especially when having in mind that in such rare events highly specific and effective intervention strategies are required to reach significance. The study reporting beneficial effects on delirium showed also a ceiling effect, with more than half of participants (65%) having no delirious day during hospitalization [26]; however, the more specific delirium-related intervention contents and the selection of a non-dichotomous, more sensitive scaling procedure for delirium (number of delirious days vs. delirious patients) might have still led to significant intervention effects. The lack of significant intervention effects documented by the MMSE [26] and the CAM [39] might also be related to their instrument type. Both were primarily developed as screening instruments, either for global cognitive functioning (MMSE) or for delirium (CAM), which may have limited the sensitivity of these instruments to detect intervention-induced changes among these two studies.

### Limitations

This review has some limitations. First, the matching procedure was based on subjective appraisals of the authors; however, standardized criteria were used which were derived from recommended guidelines [14]. To our knowledge, this review is the first to evaluate the selection of outcome measures in studies on early rehabilitation in the acute care hospital setting by such criteria, representing the most innovative feature of this review. Second, due to the international nature of this review and the inherent differences in the health care systems of the countries in which the studies were conducted, it was sometimes difficult to determine if the study took place in the acute care hospital setting. Consequently, the selection process might be affected by inconsistent terminology of the acute care hospital setting among different countries. Third, the main findings of this review were related to the primary outcome measures identified among the included studies. A clear definition of the study’s primary outcome measures in the method section of the included articles was sometimes lacking. The identification of the primary outcome measures was therefore based on the researchers’ critical appraisal of the information provided in the articles, considering especially the study aims mentioned in the articles. The identification of the primary outcome measures was also performed independently by two researches with disagreements resolved by consensus or third party consultation. Fourth, only information provided in the included articles was evaluated in this review, although the authors may have used additional or more detailed methodology not stated or unclearly described in the articles.

## Conclusions

The present systematic review provided for the first time a detailed overview and critical appraisal of the primary outcome measures used in previous RCTs to evaluate early inpatient rehabilitation for acutely hospitalized older patients. Current findings highlight that the matching of the outcome measures with especially the contents of the intervention to be evaluated represents a key factor to reveal significant benefits attributable to the intervention. Among the different categories of outcome measures, those assessing the mobility status seem to be more sensitive to intervention-induced effects of early rehabilitation programs than those assessing the functional, psychological or cognitive status, hospital outcomes, or adverse clinical events. For future studies, it is recommended to identify not only outcome measures with established psychometric properties in the different sub-samples of the acute geriatric hospital setting, but also to select outcome measures that match the specific intervention contents. Inconsistent findings on the effectiveness of early rehabilitation programs in this setting might have been partly due to the inappropriate selection of outcome measures.

## Additional files

Additional file 1: **Table S1.** Search strategy used in PubMed. (DOCX 15 kb)

Additional file 2: PRISMA checklist. (DOCX 31 kb)

Additional file 3: **Table S2.** Methodological quality scores on the PEDro scale for each included study. (DOCX 56 kb)

## Data Availability

All data were retrieved from published RCTs and extracted in Table 2. The exact references can be found in the list of references. The relevant data supporting the conclusions of this review are included within this article and its additional files.

## Abbreviations

(I)ADL: (instrumental) Activities of Daily Living
10MWT: 10-Meter Walking Test
30CST: 30-seconds Chair Stand Test
6MWT: 6-Minute Walk Test
AIS: Abbreviated Injury Scale
CAM: Confusion Assessment Method
COPD: Chronic Obstructive Pulmonary Disease
COVS: Clinical Outcome Variables Scale
FIM: Functional Independence Measure
GDS: Geriatric Depression Scale
HRQOL: Health-Related Quality of Life
ICF: International Classification of Functioning, Disability and Health
LOS: Length of stay
mDRI: Modified Disability Rating Index
mILOAS: Modified Iowa Level of Assistance Scale
mKB ADL scale: Modified Klein-Bell ADL scale
MMSE: Mini-Mental State Examination
OBS: Organic Brain Syndrome Scale
OLS: One Leg Stance
PA: Physical activity
PPAS: Self-developed postoperative patient activity scale
RCTs: Randomized controlled trials
SPPB: Short Physical Performance Battery
TUG: Timed Up and Go
UCLA scale: University of California, Los Angeles Activity scale
