# Supplementary material for: Early inpatient rehabilitation for acutely hospitalized older patients: a systematic review of outcome measures
Source: BMC Geriatr. 2019 Jul 9;19:189. doi: 10.1186/s12877-019-1201-4 (PMC6617943; doi:10.1186/s12877-019-1201-4)
Supplement: Supplementary file 1 — Table S1. Search strategy used in PubMed. (DOCX 15 kb) [file 12877_2019_1201_MOESM1_ESM.docx]

**Table S1.** Search strategy used in PubMed

| **Acute hospital setting** | **Study population** | **Early rehabilitation** | **Study design** |
| --- | --- | --- | --- |
| #1 hospitalized[tiab]  #2 “in hospital”[tiab]  #3 “acute care”[tiab]  #4 “hospital care”[tiab]  #5 inpatient*[tiab]  #6 acutely[tiab]  #7 acute[tiab]  #8 “acute setting”[tiab]  #9 “hospitalization”[Mesh;NoExp]  #10 OR (#1-#9) | #11 “geriatric patients”[tiab]  #12 aged[tiab]  #13 elder*[tiab]  #14 geriatrics[tiab]  #15 senior*[tiab]  #16 older[tiab]  #17 adult*[tiab]  #18 “older people”[tiab]  #19 “old adult*”[tiab]  #20 “older person*”[tiab]  #21 “old person*”[tiab]  #22 “aging adult*”[tiab]  #23 “aging person*”[tiab]  #24 “ageing adult*”[tiab]  #25 “humans"[Mesh]  #26 "aged/prevention and control"[Mesh]  #27 "aged/rehabilitation"[Mesh]  #28 "aged/surgery"[Mesh]  #29 "aged/therapy"[Mesh]  #30 "geriatrics/injuries"[Mesh]  #31 "geriatrics/rehabilitation"[Mesh]  #32 "geriatrics/surgery"[Mesh]  #33"geriatrics/therapy"[Mesh]  #34 "aged, 80 and over/rehabilitation"[Mesh]  #35 "aged, 80 and over/surgery"[Mesh]  #36 "aged, 80 and over/therapy"[Mesh]  #37 "frail elderly"[Mesh]  #38 OR (#11-#37)  #39 (#10 AND #38) | #40 “early rehabilitation”[tiab]  #41 “early physiotherapy”[tiab]  #42 “early mobilization”[tiab]  #43 “early mobilization”[tiab]  #44 “physical rehabilitation”[tiab]  #45 “physical functioning”[tiab]  #46 “physical function*”[tiab]  #47 “physical therapy”[tiab]  #48 “physical performance”[tiab]  #49 “physical exercise”[tiab]  #50 “physical exercises”[tiab]  #51 exercises[tiab]  #52 exercise[tiab]  #53 mobility[tiab]  #54 “early mobility”[tiab]  #55 “exercise intervention”[tiab]  #56 “exercise therapy”[tiab]  #57 “exercise therapies”[tiab]  #58 “exercise movement”[tiab]  #59 “early physical rehabilitation”[tiab]  #60 “inpatient rehabilitation” [tiab]  #61 “early physical activity”[tiab]  #62 “strength training”[tiab]  #63 “training”[tiab]  #64 “strengthening program”[tiab]  #65 “strengthening programs”[tiab]  #66 “weight bearing”[tiab]  #67 “weight lifting”[tiab]  #68 “resistance training”[tiab]  #69 “resistance program”[tiab]  #70 “exercise therapy"[Mesh]  #71 "exercise"[Mesh]  #72 "exercise movement techniques"[Mesh]  #73 "early ambulation"[Mesh]  #74 OR (#40-#73)  #75 (#10 AND #38 AND #74) | #76 "randomized controlled trial"[publication type]  #77 "controlled clinical trial"[publication type]  #78 randomized[tiab]  #79 randomly[tiab]  #80 OR (#76-#79)  #81 (#10 AND #38 AND #74 AND #80) |
