# Supplementary material for: Early inpatient rehabilitation for acutely hospitalized older patients: a systematic review of outcome measures
Source: BMC Geriatr. 2019 Jul 9;19:189. doi: 10.1186/s12877-019-1201-4 (PMC6617943; doi:10.1186/s12877-019-1201-4)
Supplement: Supplementary file 3 — Table S2. Methodological quality scores on the PEDro scale for each included study. (DOCX 56 kb) [file 12877_2019_1201_MOESM3_ESM.docx]

**Table S2** Methodological quality scores on the PEDro scale for each included study

| **Study** | **Eligibility criteria^1^** | **Randomisation^2^** | **Concealment^3^** | **Groups similar at baseline^4^** | **Blinded participants^5^** | **Blinded therapists^6^** | **Blinded assessors^7^** | **Adequate follow-up^8^** | **Intention to treat^9^** | **Between group statistics^10^** | **Point estimates and variability^11^** | **PEDro score** |
| --- | --- | --- | --- | --- | --- | --- | --- | --- | --- | --- | --- | --- |
| Abizanda 2011 | 1 | 1 | 0 | 1 | 0 | 0 | 1 | 1 | 0 | 1 | 1 | **6** |
| Asplund 2000 | 1 | 1 | 1 | 1 | 0 | 0 | 0 | 1 | 0 | 1 | 1 | **6** |
| Barnes 2012 | 1 | 1 | 0 | 1 | 0 | 0 | 0 | 0 | 0 | 1 | 0 | **3** |
| Blanc-Bisson 2008 | 1 | 1 | 0 | 1 | 0 | 0 | 0 | 0 | 1 | 0 | 0 | **3** |
| Brown 2016 | 1 | 1 | 1 | 1 | 0 | 0 | 1 | 1 | 1 | 1 | 1 | **8** |
| Counsell 2000 | 1 | 1 | 1 | 1 | 0 | 0 | 0 | 0 | 1 | 1 | 1 | **7** |
| Czyzewski 2013 | 1 | 1 | 0 | 0 | 0 | 0 | 0 | 0 | 0 | 1 | 1 | **3** |
| Eyres 2005 | 1 | 1 | 0 | 0 | 0 | 0 | 1 | 1 | 0 | 0 | 1 | **4** |
| Hagsten 2004 | 1 | 1 | 1 | 1 | 0 | 0 | 0 | 1 | 0 | 1 | 0 | **5** |
| He 2015 | 1 | 1 | 0 | 1 | 0 | 0 | 0 | 1 | 0 | 0 | 1 | **4** |
| Huusko 2000 | 1 | 1 | 1 | 0 | 0 | 0 | 0 | 1 | 0 | 1 | 1 | **6** |
| Jeffs 2013 | 1 | 1 | 1 | 1 | 0 | 0 | 1 | 1 | 1 | 1 | 1 | **8** |
| Jones 2006 | 1 | 1 | 1 | 1 | 0 | 0 | 1 | 0 | 1 | 1 | 1 | **7** |
| Kimmel 2016 | 1 | 1 | 1 | 1 | 0 | 0 | 1 | 1 | 0 | 1 | 1 | **7** |
| Landefeld 1995 | 1 | 1 | 0 | 1 | 0 | 0 | 0 | 0 | 0 | 1 | 1 | **4** |
| Naglie 2002 | 1 | 1 | 1 | 1 | 0 | 0 | 1 | 1 | 0 | 1 | 1 | **7** |
| Nikolaus 1999 | 1 | 1 | 1 | 0 | 0 | 0 | 0 | 1 | 1 | 1 | 1 | **6** |
| Oldmeadow 2006 | 1 | 1 | 0 | 1 | 0 | 0 | 1 | 1 | 0 | 1 | 1 | **6** |
| Pitkälä 2008 | 1 | 1 | 1 | 1 | 0 | 0 | 0 | 1 | 1 | 1 | 1 | **7** |
| Prestmo 2015  Taraldsen 2014 | 1 | 1 | 1 | 1 | 0 | 0 | 0 | 1 | 1 | 1 | 1 | **7** |
| Siebens et al., 2000 | 1 | 1 | 1 | 1 | 0 | 0 | 1 | 1 | 1 | 1 | 1 | **8** |
| Stenvall 2007^a,b^, 2012  Lundström 2007 | 1 | 1 | 1 | 1 | 0 | 0 | 0 | 1 | 1 | 1 | 1 | **7** |
| Torres-Sanchez 2017 | 1 | 1 | 1 | 1 | 0 | 0 | 1 | 1 | 1 | 1 | 1 | **8** |
| Vidan 2005 | 1 | 1 | 0 | 1 | 0 | 0 | 1 | 1 | 0 | 1 | 1 | **6** |
| PEDro, Physiotherapy Evidence Database, 1: criterion is evidenced in the article; 0: criterion is not evidenced, not applicable, not coded, or could not be determined in the article. ^1^ Eligibility criteria were specified [Note: Item is not used to calculate PEDro score];^2^Participants were randomly allocated to groups; ^3^Allocation to groups was concealed; ^4^The groups were similar at baseline regarding the most important prognostic indicators; ^5^Participants were not aware of the group in which they were allocated (blinded); ^6^Staff that administered training was not aware (blind) of the group status (intervention-control); ^7^Assessors measuring at least one key outcome were not aware (blind) of the group status; ^8^Measures of at least one key outcome were obtained from more than 85% of the subjects initially allocated to groups; ^9^All subjects for whom outcome measures were available received treatment or control condition as allocated or, where this was not the case, data for at least one key outcome were analysed by “intention to treat”; ^10^The results of between-group statistical comparison are reported for at least one key outcome; ^11^The study provides both point measures and measures of variability for at least one key outcome. | | | | | | | | | | | | |
